# Enablers of the successful implementation of simulation exercises: a qualitative study among nurse teachers in undergraduate nursing education

**DOI:** 10.1186/s12912-021-00756-3

**Published:** 2021-11-22

**Authors:** Kristine Haddeland, Åshild Slettebø, Mariann Fossum

**Affiliations:** Centre for Caring Research – Southern Norway, Faculty of Health and Sport Sciences, University of Agder, Postbox 422, 4604 Kristiansand, Norway

**Keywords:** Clinical deterioration, Interview, Nursing education, Simulation training, Teaching method

## Abstract

**Background:**

Simulation exercises are increasingly being used as a teaching method in the field of undergraduate nursing education. Thus, the present study sought to identify, describe and discuss enablers of the successful implementation of simulation exercises in undergraduate nursing education.

**Methods:**

This study had a qualitative descriptive design and involved individual interviews conducted between November and December 2018 with six nurse teachers from three different university campuses in Norway. The transcribed interviews were analysed by means of a qualitative thematic analysis.

**Results:**

The majority of the interviewees wanted to offer more simulation exercises as part of their respective undergraduate nursing education programmes. Moreover, creating a safe environment, facilitating student-centred learning and promoting reflection were all identified by the interviewees as enablers of the successful implementation of simulation exercises.

**Conclusions:**

The findings of this study indicate that nurse teachers consider simulation to be a valuable teaching method for improving students’ learning outcomes. In addition, the findings could guide the future implementation of simulation exercises in undergraduate nursing education.

**Trial registration:**

ClinicalTrials.gov ID: NCT 04063319.

Protocol ID: 52110 Nursing Students’ Recognition of and Response to Deteriorating Patients.

## Introduction

Clinical nurses face many physical and psychological stressors in the workplace, including the need to cope with limited resources in clinical practice [1]. At the same time, there is increasing recognition of the need to improve both care quality and patient safety, with the increased focus on patient safety leading to a decrease in the number of clinical placement opportunities available for nursing students [2]. This decrease in placement opportunities presents learning challenges and has implications for nursing education due to limiting students’ hands-on experience in acute care situations [3, 4]. In fact, it has been found that newly qualified nurses do not have the crucial skills and understanding concerning nursing practice that are expected by leaders in clinical practice [5]. However, the increased use of simulation exercises in undergraduate nursing education could represent an effective strategy for addressing this educational gap [6].

According to the International Nursing Association for Clinical Simulation and Learning’s (INACSL’s) Standards of Best Practice in Simulation [7], the concept of simulation can be defined as ‘an educational strategy in which a particular set of conditions are created or replicated to resemble authentic situations that are possible in real life’ (p. 44). Clinical simulation can be delivered using different modalities, including actors, standardised patients and human patient simulators [8]. To enhance students’ learning from simulation exercises, the INACSL’s Standards of Best Practice in Simulation [9] suggest that simulation exercises include three phases: (a) a briefing phase, (b) a scenario phase and (c) a debriefing phase (p. 8). During the briefing phase, the main aim is to establish a psychologically safe environment for the participants [9, 10]. Next, the scenario phase provides the participants with an opportunity to achieve identified objectives in a simulated reality [9, 10]. The final phase of the simulation exercise, the debriefing phase, involves a reflective process whereby feedback is provided regarding the participants’ performance during the exercise [9, 10].

A number of studies have shown that undergraduate nursing students’ knowledge and confidence are increased after participating in simulation exercises [11–14]. Moreover, students have expressed satisfaction with simulation as a teaching method [14–16]. However, although the literature describes multiple methods for facilitating simulated scenarios, less is known about nurse teachers’ experiences of organising and using simulation exercises [10, 17]. Thus, the present study sought to identify, describe and discuss enablers of the successful implementation of simulation exercises in the field of undergraduate nursing education.

## Methods

### Design

This study had a qualitative descriptive design and involved individual interviews conducted with nurse teachers working in undergraduate nursing education. The study was conducted in accordance with the Consolidated Criteria for Reporting Qualitative Research (COREQ) checklist [18].

### Participants and settings

A convenience sample (*n* = 6) of nurse teachers from three campuses of two universities were invited by email to participate in the study between November and December 2018. Two of the campuses were located in southern Norway. (Campus A and Campus B), while the third was in eastern Norway (Campus C). The invited nurse teachers were chosen because they were involved in organising and using one specific simulation exercise in their respective undergraduate nursing programmes, and because they were involved in all of the simulation phases (briefings, scenarios and debriefings). They all responded positively to the invitation to participate in the study. The simulation exercise took place in two simulation laboratories at Campus A and Campus C. A total of 140 s-year undergraduate nursing students participated in the study (Campus A = 72, Campus B = 50 and Campus C = 18). The students were divided into 13 simulation groups, with each group comprising 8–15 participants. The human patient simulator Laerdal SimMan 3G was used in all of the scenarios, and a total of two nurse teachers were actively involved in each simulation group. The simulation exercise lasted for approximately 2 h. Additional information concerning the simulation exercise is available in Table 1.

**Table 1:** Information regarding the simulation exercise used in the study [11]

Learning objective:	Recognise and respond appropriately to acute patient deterioration (hypovolemia).
Simulation deliverers:	A total of seven nurse teachers were involved in organising the simulation exercise.
	One operator and one facilitator were present in each simulation group.
Patient information:	A 75-year-old female patient hospitalised with cancer. She has gone through surgery (hemicolectomy) and been moved to the surgical ward.
Simulation phases:	1. Briefing (60 min):
	- Recalling the patient information and the learning objective
	- Selection of an active participant or observer role.
	2. Scenario (15 min)
	- Working through the scenario (with support from a nurse teacher if required).
	3. Debriefing (45 min)
	- Participating in a debriefing structured into four phases: reaction, description, analysis and application.
Roles in the scenario:	Registered nurse × 2, relative, physician and observers

### Data collection

The participating nurse teachers were asked to share their experiences regarding organising and using the simulation exercise. An interview guide consisting of five open-ended questions was prepared for use in the study. The questions were as follows: *What role did you play in organising the simulation exercise? How would you describe your experience with the simulation exercise? What were the positive points of the simulation exercise? What were the negative points of the simulation exercise? Do you have something to add regarding the simulation exercise?* In addition, several follow-up questions (e.g. *Can you elaborate on that? How did you experience it?*) were also used. Prior to the data collection, the interview guide was pilot tested on a nurse teacher with experience of organising simulation exercises. All of the interviews were audio recorded, and they lasted from 17 to 48 min (mean: 33 min). Each interview was conducted by the first author at the university where the relevant simulation exercise took place, and all of the interviews were conducted within 1 week after the final simulation exercise was arranged. Only the first author and the relevant participant were present at each interview.

### Data analysis

First, the interviews were all transcribed verbatim by the first author. To ensure familiarisation with the data, the transcribed text was repeatedly read. A six-step thematic analysis based on the work of Braun and Clarke [19] was performed to identify the codes, sub-themes and themes within the data. The six steps were: 1) ensuring familiarisation with the data, 2) generating the initial codes, 3) searching for themes, 4) reviewing the themes, 5) defining and naming the themes and 6) producing the report [19]. The obtained themes and sub-themes were reviewed by all of the authors. Three examples of the thematic analysis are presented in Table 2.

**Table 2:** Three examples of the qualitative thematic analysis

Examples of text coded	Sub-theme	Theme
‘I know that some of the nursing students have limited prior experience with simulation, and I understand that there are a lot of new impressions for them to take in. It takes some time to feel safe and comfortable, so I think my most important role is to make them safe before the scenario.’ (Participant 2)	Preparing for the scenario	Creating a safe environment
‘All of the simulation groups and participants are different, so it is difficult to follow a very strict procedure.’ (Participant 6)	Simulation exercises should meet students’ needs	Facilitating student-centred learning
‘I think it’s very good that the participants’ come up with this during the debriefing sessions, that there are actually things they thought they should have done differently in the scenario. Then I think, “Very good!” Then there has been a learning process and it is very valuable.’ (Participant 1)	Facilitate participants learning through reflection	Promoting reflection

## Results

### Participants’ demographics

The participants included five females and one male. They ranged in age from 39 to 64 years (md: 46). All of them had previous experience of organising and using simulation exercises for nursing students in undergraduate nursing education programmes. Their years of experience ranged from 1.5 years to 15 years. All of the participants except for one had attended a course that focused on how to act as a facilitator in relation to simulation exercises. Moreover, two of them had also been involved in organising facilitator courses. There were two participants from each university campus. Three of them were facilitators in the scenarios, one was an operator, while the remaining two nurse teachers acted as both a facilitator and an operator due to being involved in more than one scenario during the data collection period.

### Themes

Three main themes were identified from the interview data: (1) creating a safe environment, (2) facilitating student-centred learning and (3) promoting reflection. In the subsections below, quotes from the interviews are included in the presentation of the themes to better illustrate the meaning of the text.

#### Creating a safe environment

Feeling secure, especially at the beginning of the exercise, was reported to be a key aspect of the learning process. Several factors were reported to help create a safe environment before, during and after the scenario. Providing the students with information a week before the simulation exercise and starting the briefing phase by reviewing the patient’s case, setting out the objectives, repeating the relevant theoretical knowledge and agreeing on terms of mutual respect and confidentiality were all highlighted as important. In fact, mutual respect and confidentiality within the simulation groups were frequently referenced. As one participant stated:

*Before the scenario, I talk about confidentiality for everyone who participates ( … ). I also highlight that they should not laugh at each other, that they should be kind to each other, and that they should offer constructive feedback. I establish it as a frame for the whole simulation exercise before we start. (Participant 5)*

Talking with all of the students about their roles and providing a thorough introduction to the patient simulator prior to starting the scenario were also said to be important. One participant reported:

*It is important that everyone gets to touch the patient simulator and feel its pulse rate. They can also see that the patient’s chest rises. And then we show them the equipment that could be used during the scenario. (Participant 4)*

The importance of assigning everyone in the simulation group a responsibility was emphasised, and the observers were also assigned tasks to focus on. During the scenario, the facilitators were present in the simulation room and offered practical assistance or cues if required. They emphasised the need for technical expertise in terms of managing the patient simulator, and they stressed that technical errors during the scenario could make the students feel insecure. The nurse teachers perceived that the students found it uncomfortable being observed by the other participants in the simulation group during the scenario, although that feeling reduced when they began concentrating on what they had to do. As one participant commented:

*She said she was very concerned at first that she was being observed, but then she just decided that she had to stop thinking about it and move on. (Participant 4)*

All of the participants reflected on the appropriate time to end the scenario. Several of the participants commented that they preferred to end the scenario at a point at which the students had managed something and the situation was clarified, which they felt made the students feel more secure and gave them a sense of achievement. One participant said:

*I think it’s important to end the scenario when things are positive and the students have managed something. We should not end it in the middle of something that the students feel to be dramatic. (Participant 6)*

With regard to the debriefing phase, the participants also emphasised the importance of focusing on the positive aspects of what the students had done to make them feel more secure.

### Facilitating student-centred learning

The participants reported that simulation exercises should be student-centred and tailored to meet students’ needs. Recognising the students’ level of knowledge was reported to be important. To ensure that the exercise was pitched at the right level of knowledge, half of the participants felt that the students should be active during most of the briefing phase. One participant reported:

*There is always a question when preparing the briefing phase: how much information should the students receive? The more advanced the information is that you give them before the scenario, I have noticed that they become more stressed. Therefore, rather than give them information, I ask them to share their knowledge as much as possible to strengthen their belief that they can manage the scenario. I think it is very important that the participants think* ‘*we have a lot of knowledge and now we will try to use it’ before starting the scenario. (Participant 5)*

Not knowing how the students would respond during the scenario and potentially being unprepared for new aspects that might arise made organising the simulation exercise unpredictable. The participants who acted as operators in the scenarios pointed out that sometimes they had to give the patient more symptoms than they had planned to do and possibly even more than was realistic (such as higher blood pressure or reduced awareness) to prompt the students to respond. As one participant reported:

*One must be prepared to respond to all of the possible actions that the students do or not do. (Participant 5)*

The debriefing phases were recognised as being particularly unpredictable because they were based on what the students highlighted. One participant said:

*In the debriefing phase, the students highlight what they want to emphasise from the scenario, so it can take slightly different forms based on what they share. (Participant 3)*

To reduce the unpredictability, the participants relied on the fact that they were two professionals who shared the organisation of the simulation exercise. They made appointments before each scenario started, communicated during the scenario if necessary and supported each other throughout all of the simulation phases. The participants also acknowledged the value of supporting each other after the simulation exercise had finished. One participant expressed the matter as follows:

*We talk a lot afterwards about how the simulation exercises have been, and it serves as a kind of colleague guidance as well. (Participant 3)*

This kind of colleague guidance was reported to be especially important in relation to coping better with unexpected situations that may arise during simulation exercises in the future.

### Promoting reflection

The third theme concerned the high value that the participants placed on promoting reflection. The participants particularly highlighted the importance of learning from reflection through analysis and discussion within the simulation group immediately after the scenario is completed. As one participant said:

*Being able to see it from someone else’s perspective, rather than just your own, is important. (Participant 1)*

The participants noted that the students who played active roles reported finding the scenario to be chaotic and making many mistakes. Therefore, the chance to share different perspectives on the situation within the simulation group immediately after finishing the scenario was highly appreciated. One participant commented:

*After the scenario, it is always a bit like this: “Oh no!” It's never like this: “Yes, this went well!” ( … ) The students are quick to point out everything they did wrong ( … ). Therefore, I ask them to first talk about something that they did well, to try to get them to change focus. (Participant 2)*

The participants emphasised that the debriefing phase should be tailored to the learning objectives. They recognised that starting the debriefing phase by asking about the students’ emotional situation could shift the focus away from the learning outcomes, which meant that they did not dwell on the emotional side of participating. One participant reflected:

*If you start by asking too much about emotions in the beginning, then you can have some problems getting further in the debrief ( … ). I write the learning objectives on a screen in the room, and I read them aloud to everybody and say: “In relation to these learning objectives, can you describe something you did that you think was of a high quality?” (Participant 4)*

To enhance the learning outcomes and promote reflection, the participants emphasised the importance of allowing more students to take on active roles during the scenarios. To manage this, they suggested repeating the scenarios in each simulation group or splitting the scenarios with breaks during which the students changed roles. They thought that having more students play active roles would result in more students having a sense of achievement. Furthermore, it would make it easier for them to get to know each other better within the simulation group, make them feel more secure and probably prompt them to be more honest when sharing their reflections.

The majority of the participants wanted to offer more simulation exercises as part of their respective undergraduate nursing education programmes, both before and during students’ clinical practice periods. One participant reported:

*One can imagine that the students would have had more to contribute if they had spent some days in clinical practice before the simulation exercise. They should have a follow-up simulation exercise after they have been out in the clinical environment a bit and gained a little more experience. (Participant 5)*

The value of simulation exercises that were tailored to students’ own clinical experiences was highlighted as helping to improve their reflections and learning outcomes.

## Discussion

The present study sought to identify, describe and discuss enablers of the successful implementation of simulation exercises in the field of undergraduate nursing education. The findings highlight the importance of nurse teachers’ role in creating a safe environment, facilitating student-centred learning and promoting reflection to increase the students’ learning outcomes from simulation exercises. Furthermore, the findings indicate that the participating nurse teachers consider simulation to be a valuable teaching method for improving students’ learning, which is in line with the findings of prior studies [20, 21].

The participants elaborated on the need to create a safe environment in order to improve the outcomes of simulation exercises. The importance of having tailored knowledge prior to the scenario was highlighted, which could prove helpful in enhancing learning during simulation exercises [22]. If students are informed about the scenario and the associated learning objectives in advance, they will have the opportunity to refer to theory and perhaps be more easily able to reflect on what specific types of actions may be expected of them [23]. It is evident that students who participate in briefing activities that include orientation tasks perceive themselves to exhibit higher efficacy in simulation exercises [24], while the importance of orientation concerning the simulation environment prior to the scenario was highlighted in a Delphi study regarding quality enablers in relation simulation exercises [25]. The highlighted importance of nurse teachers’ role during the briefing phase, that is, describing the learning objectives and equipment used in the scenario, is also in line with the findings of other studies [9, 10].

However, the nurse teachers in the present study highlighted the need to not overload the students with too much information during the orientation. The patient simulator offers a variety of options, and if completing the exercise feels unattainable for the students, they may become increasingly insecure before the scenario. Beischel [26] found that students who spent more than an hour on briefings were more nervous before the scenario when compared with those who spent less time preparing for simulation exercises. In addition, Cuerva et al. [27] found that the learning outcomes were better following a short orientation and an abrupt start to the scenario, rather than a long briefing session that includes direct instruction concerning the scenario.

The findings of this study highlighted that mutual respect and confidentiality among members of the simulation groups were important. Trust is a key responsibility of nurse teachers when it comes to creating and maintaining a safe environment for simulation exercises [9, 10]. The findings of this study also indicated that some students found it uncomfortable being observed by other participants in the simulation group during the scenario. This finding is in line with the findings of other studies [28, 29], although Kelly et al. [30] found that being observed ranked low in terms of what was considered most important in simulation exercises. Trokan-Mathison [31] indicated that being observed by others during simulation exercises was less stressful than being observed in clinical practice.

Facilitating student-centred learning was identified as another theme in the present study. To ensure that students’ needs are met, the participants stressed that they should be active most of the time during simulation exercises. This is in accordance with the findings of Dieckmann [32], who suggested that allowing participants to do most of the talking during the debriefing phase boosted their efficacy. However, not knowing how the students would react or what aspects of the scenario they would highlight during the debriefing phase made organising the simulation intervention unpredictable for the nurse teachers in this study. In terms of reducing the unpredictability, the participants recognised the value of being two professionals who shared the organisation of the simulation exercise and had the opportunity to swap colleague guidance afterwards. Davis et al. [21] found that nurse teachers were often insecure with regard to the technology aspect of the simulation exercises, as they feared that they would not know how to use the technology during the scenarios. Colleague support and guidance before, during and after organising simulation exercises may help to reduce this fear. The importance of staff training in relation to simulator technology and scenario design has previously been identified as essential and should be considered an important aspect when organising simulation exercises [25].

Promoting reflection was identified as another important theme in this study. According to Dieckmann [32], reflection occurs explicitly before and after the scenario but also implicitly during the scenario. The importance of promoting reflection and learning through sharing different perspectives on the situation immediately after the scenario has been identified in other studies in the field of nursing [25, 33–35]. Kaldheim et al. [33] identified how theatre nursing students found it easier to capture non-technical skills when acting as an observer and, further, how observing others was essential in terms of gaining competence in communication, interdisciplinary collaboration and prioritisation in acute situations. In the present study, the debriefing phase followed the Promoting Excellence and Reflective Learning in Simulation (PEARLS) framework [36], as recommended by the INACSL [37]. Research has identified that the means by which educators facilitate debriefing phases vary greatly [38] and that novice instructors who use a debriefing script are more effective at increasing learners’ knowledge acquisition than educators who did not use a script [39]. Moreover, a comparison of debriefing methods identified that nursing students who received facilitated debriefing achieved higher scores on the subsequent simulation when compared with students who only received feedback or self-debriefing [40].

By using the PEARLS framework in the present study, the nurse teachers found that asking about the students’ emotional situation could shift the focus away from the learning outcomes. Cheng et al. [41] reported that students should share their initial reactions to avoid having unresolved negative emotions that may decrease their learning outcomes. The authors observed that students’ emotions, such as anger, frustration and anxiety, are often missed or ignored during the debriefing phase [41]. Husebø et al. [42] found that mostly evaluative questions and a few emotional questions are asked in the debriefing phase. Gibbs’s reflective cycle [43] is another theoretical framework for guiding the debriefing phase that includes the emotional dimension. It includes six stages: description, feelings, evaluation, analysis, conclusion and action plan [43]. In line with the work of Eppich and Cheng [36], Gibbs [43] agreed that if emotions are not dealt with adequately, students may return to emotions associated with the simulation experience at a later stage in the debriefing phase when they should be considering implications and actions. Focusing on students’ feelings as stage number two in the debriefing phase when using Gibbs’s reflective cycle [43] could represent a better way of structuring the debriefing phase.

The participants in the present study suggested that more simulation exercises should be included in nursing education both before and during students’ clinical practice periods. In addition, they highlighted how the simulation exercises should be tailored to the students’ own practical experiences to help improve their learning. The number of rehearsals required to manage, for example, measuring patients’ vital signs appropriately may be impossible to achieve in the unpredictable environment of clinical practice. Thus, simulation exercises wherein multiple rehearsals are available may enable nursing students to gain necessary skills more rapidly than if left to learn solely through clinical practice [44].

### Strengths and limitations

The present study had a number of limitations. First, the study involved a small sample. Nevertheless, the findings provide an understanding of nurse teachers’ experiences of using simulation exercises as a teaching method, which may provide valuable guidance for the future implementation of simulation exercises in the field of undergraduate nursing education. There were also numerous congruent findings during the data collection, which may be an indication of saturation.

Interpretation is influenced by researchers’ preconceptions. All of the authors are female nurse teachers with expertise in qualitative analysis. The authors were not involved as teachers of the students who participated in the simulation exercise. However, relationships between the authors and four of the participants were established prior to the data collection because they worked at the same university. The first author, who conducted all of the interviews, had no previous experience of organising simulation exercises, which could be viewed as a strength in this setting. All of the participants were aware that the first author conducted the interviews as part of a doctoral dissertation. It was also considered a strength that the same interview guide was used for all of the participants and, further, that the same researcher conducted all of the interviews.

## Conclusions

Creating a safe environment, facilitating student-centred learning and promoting reflection were all identified by the nurse teachers in this study as important enablers of the successful implementation of simulation exercises in the field of undergraduate nursing education. Most of the nurse teachers wanted to include more simulation exercises in their respective undergraduate nursing education programmes. In addition, the present study can provide valuable guidance for the future implementation of simulation exercises.

## Data Availability

The datasets generated and analysed in the present study are not publicly available due to concerns that participants’ privacy may be compromised; however, they are available from the corresponding author upon reasonable request.

## Abbreviations

INACSL: International Nursing Association for Clinical Simulation and Learning
COREQ: Consolidated Criteria for Reporting Qualitative Research
PEARLS: Promoting Excellence and Reflective Learning in Simulation

## References

[1] Akbar RE, Elahi N, Mohammadi E, Khoshknab MF. How do the nurses cope with job stress? A study with grounded theory approach. J Caring Sci. 2017. 10.15171/jcs.2017.020. 10.15171/jcs.2017.020 PMC5618945 28971071

[2] Dahlgren MA, Felländer-Tsai L, Nyström S, Rystvedt H, Dahlgren MA, Rystvedt H, Felländer-Tsai L, Nyström S (2019). Why this book?. Interprofessional simulation in health care.

[3] Lee AH, Kelley C, Alfes CM, Bennington LK, Dolansky MA. High-fidelity patient simulation to evaluate student nurse patient safety competency. Clin Simul Nurs. 2017. 10.1016/j.ecns.2017.08.006.

[4] Shin S, Jin-Hwa P, Jung-Hee K (2015). Effectiveness of patient simulation in nursing education: meta-analysis. Nurse Educ Today.

[5] Burgess A, Buc HB, Brennan JM (2015). Using a complex patient management scenario to help bridge the education-practice gap. Nurs Educ Perspect.

[6] Huston CL, Phillips B, Jeffries P, Todero C, Rich J, Knecht P, Sommer S, Lewis MP (2018). The academic-practice gap: strategies for an enduring problem. Nurs Forum.

[7] INACSL (2016). Standards Committee INACSL standards of best practice: simulation Simulation glossary. Clin Simul Nurs.

[8] Stayt LC, Merriman C, Ricketts B, Morton S, Simpson T (2015). Recognizing and managing a deteriorating patient: a randomized controlled trial investigating the effectiveness of clinical simulation in improving clinical performance in undergraduate nursing students. J Adv Nurs.

[9] INACSL (2016). Standards Committee INACSL standards of best practice: simulation Simulation design. Clin Simul Nurs.

[10] Kelly M, Guinea S, Nestel D, Kelly M, Jolly B, Watson M (2018). Facilitating healthcare simulations. Healthcare simulation education. Evidence, theory and practice.

[11] Haddeland K, Slettebø Å, Svensson E, Tosterud R, Wangensteen S, Fossum M. The Effects of Using High-Fidelity Simulation in Undergraduate Nursing Education: A Multicenter Randomized Controlled Trial with a Process Evaluation. Int J Educ Res. 2021;109:101813. 10.1016/j.ijer.2021.101813.

[12] Mulyadi M, Tonapa SI, Rompas SSJ, Wang R-H, Lee B-O (2021). Effects of simulation technology-based learning on nursing students' learning outcomes: a systematic review and meta-analysis of experimental studies. Nurse Educ Today.

[13] Labrague LJ, McEnroe-Petitte DM, Bowling AM, Nwafor CE, Tsaras K (2019). High-fidelity simulation and nursing students’ anxiety and self-confidence: a systematic review. Nurs Forum.

[14] Cant RP, Cooper SJ (2017). Use of simulation-based learning in undergraduate nurse education: an umbrella systematic review. Nurse Educ Today.

[15] Hustad J, Johannessen B, Fossum M, Hovland OJ (2019). Nursing students’ transfer of learning outcomes from simulation-based training to clinical practice: a focus-group study. BMC Nurs.

[16] Warren JN, Luctkar-Flude M, Godfrey C, Lukewich J (2016). A systematic review of the effectiveness of simulation-based education on satisfaction and learning outcomes in nurse practitioner programs. Nurse Educ Today.

[17] Krogh K, Bearman M, Nestel D (2016). “Thinking on your feet” – a qualitative study of debriefing practice. Adv Simul.

[18] Tong A, Sainsbury P, Craig J (2007). Consolidated criteria for reporting qualitative research (COREQ): a 32-item checklist for interviews and focus groups. Int J Qual Healthc.

[19] Braun V, Clarke V (2006). Using thematic analysis in psychology. Qual Res Psychol.

[20] Tjoflåt I, Koyo SL, Bø B. Simulation-based education as a pedagogic method in nurse education programmes in sub-Saharan Africa – perspectives from nurse teachers. Nurse Educ Pract. 2021. 10.1016/j.nepr.2021.103037. 10.1016/j.nepr.2021.103037 33839595

[21] Davis AH, Kimble LP, Gunby SS (2014). Nursing faculty use of high-fidelity human patient simulation in undergraduate nursing education: a mixed-methods study. J Nurs Educ.

[22] Paige JB, Daley BJ (2009). Situated cognition: a learning framework to support and guide high-fidelity simulation. Clin Simul Nurs.

[23] Onda EL (2012). Situated cognition: its relationship to simulation in nursing education. Clin Simul Nurs.

[24] Chamberlain J (2015). Prebriefing in nursing education: a concept analysis using Rodger’s methodology. Clin Simul Nurs.

[25] Arthur C, Levett-Jones T, Kable A (2013). Quality indicators for the design and implementation of simulation experiences: a Delphi study. Nurse Educ Today.

[26] Beischel KP (2013). Variables affecting learning in a simulation experience: a mixed methods study. Western J Nurs Res.

[27] Cuerva M, Piñel C, Martin L, Espinosa J, Corral OJ, Mendoza N. Teaching childbirth with high-fidelity simulation. Is it better observing the scenario during the briefing session? J Obstet Gynaecol. 2018. 10.1080/01443615.2017.1393403. 10.1080/01443615.2017.1393403 29433368

[28] Nielsen B, Harder N (2013). Causes of student anxiety during simulation: what the literature says. Clin Simul Nurs.

[29] DeCarlo D, Collingridge D, Grant C, Ventre K (2008). Factors influencing nurses’ attitudes toward simulation-based education. Simul Healthc.

[30] Kelly MA, Hager P, Gallagher R (2014). What matters most? Students’ rankings of simulation components that contribute to clinical judgment. J Nurs Educ.

[31] Trokan-Mathison N (2013). Using simulation to foster the quality and safety education for nurses’ competencies in associate degree nursing students [doctoral dissertation].

[32] Dieckmann P (2009). Using simulations for education, training and research.

[33] Kaldheim HKA, Fossum M, Munday J, Creutzfeldt J, Slettebø Å (2021). Use of interprofessional simulation-based learning to develop perioperative nursing students’ self-efficacy in responding to acute situations. Int J Educ Res.

[34] Rogers B, Baker KA, Franklin AE (2020). Learning outcomes of the observer role in nursing simulation: a scoping review. Clin Simul Nurs.

[35] Husebø SE, O’Regan S, Nestel D (2015). Reflective practice and its role in simulation. Clin Simul Nurs.

[36] Eppich W, Cheng A (2015). Promoting excellence and reflective learning in simulation (PEARLS). Simul Healthc.

[37] INACSL (2016). Standards Committee INACSL standards of best practice: simulation. Debriefing Clin Simul Nurs.

[38] Tannenbaum SI, Cerasoli CP (2013). Do team and individual debriefs enhance performance?. A meta-analysis Human Fact.

[39] Cheng A, Hunt EA, Donoghue A, Nelson-McMillan K, Nishisaki A, Lefore J, et al. Examining pediatric resuscitation education using simulation and scripted debriefing. A multicenter randomized trial. JAMA Pediatr. 2013. 10.1001/jamapediatrics.2013.1389. 10.1001/jamapediatrics.2013.1389 23608924

[40] Gantt L, Overton S, Avery J, Swanson M, Elhammoumi CV (2018). Comparison of debriefing methods and learning outcomes in human patient simulation. Clin Simul Nurs.

[41] Cheng A, Grant V, Robinson T, Catena H, Lachapelle K, Kim J, Adler M, Eppich W (2016). The promoting excellence and reflective learning in simulation (PEARLS) approach to health care debriefing: a faculty development guide. Clin Simul Nurs.

[42] Husebø SE, Dieckmann P, Rystvedt H, Søreide E, Friberg F (2013). The relationship between facilitators’ questions and the level of reflection in postsimulation debriefing. Simul Healthc.

[43] Gibbs G (1988). Learning by doing. A guide to teaching and learning methods.

[44] Stayt LC (2012). Clinical simulation: a sine qua non of nurse education or a white elephant?. Nurse Educ Today.

[45] World Medical Association. Declaration of Helsinki. Ethical principles for medical research involving human subjects. JAMA. 2013. 10.1001/jama.2013.281053. 10.1001/jama.2013.281053 24141714

